# Impact of Processing and Char Feedstock on the Thermal, Mechanical, and Electrical Behavior of PLLA Composites

**DOI:** 10.3390/polym18070871

**Published:** 2026-04-01

**Authors:** Donatella Duraccio, Boutheina Rzig, Mattia Di Maro, Giulio Malucelli, Finizia Auriemma, Federica Pignatelli, Giuliana Magnacca, Pier Paolo Capra, Mattia Bartoli, Maria Giulia Faga

**Affiliations:** 1 Istituto di Scienze e Tecnologie per l’Energia e la Mobilità Sostenibili, Consiglio Nazionale delle Ricerche, Strada delle Cacce 73, 10135 Turin, Italy; 2 Dipartimento di Scienza Applicata e Tecnologia, Politecnico di Torino, Corso Duca degli Abruzzi 24, 10123 Turin, Italy; 3 Dipartimento di Chimica “Paolo Corradini”, Università di Napoli “Federico II”, Complesso Monte S. Angelo, Via Cintia, 80126 Naples, Italy; 4 Dipartimento di Chimica, Università di Torino, Via P. Giuria 7, 10125 Turin, Italy; 5 Istituto Nazionale di Ricerca Metrologica, Strada delle Cacce 91, 10135 Turin, Italy; 6 Istituto Italiano di Tecnologia-Center for Sustainable Future Technologies, Via Livorno 60, 10144 Turin, Italy; 7 Consorzio Interuniversitario Nazionale per la Scienza e Tecnologia dei Materiali (INSTM), Via G. Giusti 9, 50121 Florence, Italy

**Keywords:** PLLA, biochar, char, composites, pyrolysis, mechanical properties, electrical conductivity

## Abstract

This work explores the influence of two preparation methods, solvent casting and melt mixing, on the structure–property relationships of poly-L-lactic acid (PLLA) composites reinforced with char derived from different waste feedstocks. Three types of char were produced by slow pyrolysis at 550 °C: olive pruning waste biochar (OC), tyre-derived char (TC), and a 1:1 hybrid co-pyrolyzed char (OTC). Each filler was incorporated into PLLA at 1 and 2 wt.% loadings, and the resulting composites were characterized through physicochemical, thermal, mechanical, and electrical analyses. Raman, FTIR, and SEM analyses revealed distinct structural characteristics for each char, with the hybrid OTC exhibiting the highest structural order due to synergistic interactions during co-pyrolysis. The preparation method affected filler dispersion. Solvent-cast films displayed micrometric agglomerates and interfacial voids, whereas melt mixing ensured a more homogeneous distribution. Thermal characterization showed that char addition did not significantly alter the crystallization or melting behavior of PLLA, although melt-mixed samples exhibited restricted chain mobility. Mechanical tests revealed opposing effects of filler loading depending on processing: in solvent-cast materials, stiffness increased while strength remained nearly unaffected, whereas melt-mixed composites exhibited reduced modulus and strength, attributed to the disruption of the denser amorphous structure generated during melt processing. Electrical resistivity depended on the preparation method. Solvent-cast composites remained insulating, while melt mixing, with OTC at 2 wt.%, led to a resistivity drop (down to 0.02 × 10^15^ Ω·cm from 20 × 10^15^ Ω·cm for unfilled PLLA), although all materials remained within the insulating regime. Overall, this work provides insight into the role of sustainable char fillers in improving the performance of PLLA composites and highlights the interplay between processing method and material properties. The developed PLLA/char composites are promising candidates for applications in flexible electronics, sensors, and antistatic components, as well as in lightweight structural materials and energy devices.

## 1. Introduction

The development of high-performance, bio-based polymer composites is increasingly driven by the need to reduce environmental impact while meeting the functional demands of advanced materials applications. Poly(lactic acid) (PLA) is a material of significant interest due to its renewable origin and a wide spectrum of applications in medicine, packaging, agriculture, electronics, and the automotive industry [1,2,3]. However, its relatively modest thermal stability, electrical insulation, and mechanical performance often limit its broader utilization. Incorporating carbon-rich fillers derived from waste streams is an attractive strategy to enhance PLA’s properties while simultaneously valorizing low-value byproducts. Char derived from pyrolysis is a versatile, carbon-rich material obtained through the thermal decomposition of organic feedstocks in an oxygen-limited environment. Depending on the source material, pyrolytic char exhibits distinct physicochemical characteristics that make it a valuable functional filler in polymer composites [4,5]. However, the effectiveness of such fillers depends critically on both their intrinsic characteristics and the processing strategies used to disperse them within the polymer matrix.

Processing method is a key determinant of composite microstructure, influencing filler dispersion, interfacial interactions, and ultimately the resulting property profile. In particular, solvent casting—a low-shear, solution-based method—and melt mixing—a high-shear, thermomechanical approach—can yield markedly different composite morphologies and structure-property relationships. Understanding how these techniques affect the performance of PLA composites containing char fillers is essential for guiding scalable, application-oriented material design. Alternative methods, such as in situ polymerization or electrospinning, generally require more complex setups, offer limited scalability, or yield materials with restricted sample sizes, making them less suitable for systematic investigation [6,7,8].

Arrigo et al. [9] explored the thermomechanical behavior of PLA-based composites incorporating biochar derived from spent ground coffee, using two distinct fabrication techniques: melt mixing and solvent casting. Their findings revealed that the processing route significantly influenced the thermal characteristics of the composites, with biochar incorporation leading to a reduction in thermal stability. Similarly, Hernandez-Charpak et al. [10] examined the impact of two biochar feedstocks (dairy manure and wood chips) on the morphological, thermal, and mechanical properties of various polymer matrices, including PLA. Their results demonstrated that both the type of biochar and the nature of the polymer matrix play a critical role in defining the overall material performance, especially in terms of mechanical strength and thermal resistance. Infurna et al. [11] investigated how biochar produced from the anaerobic digestion of the organic fraction of municipal solid waste influences the mechanical, rheological, and morphological behavior of PLA/PBAT (poly(butylene adipate-co-terephthalate)) blends. In a related study, Papadopoulou et al. [12] demonstrated that introducing small amounts of biochar derived from pelleted miscanthus straw (1, 2.5 and 5 wt.%) into poly(butylene succinate) led to significant improvements in both tensile and impact strength. Additionally, such biochar was shown to enhance UV resistance [13]. These enhancements can result from the direct effects of the filler, as well as from such indirect effects as modifications to the crystallinity of the polymer. Filler dispersion and processing technique are known to play crucial roles in dictating structural homogeneity, crystallinity, and functional efficiency [14]. However, while numerous studies have confirmed the potential of char to enhance the thermal, mechanical and electrical properties of polymers, thereby improving sustainability and reducing the overall cost of composite production [5,15], few studies have investigated the combined influence of processing methods and char types on the performance of the final composite.

In this work, we examine the influence of processing method on PLA composites reinforced with three char types obtained from distinct waste resources: olive-pruning waste char (OC), end-of-life tyre char (TC), and a hybrid olive-tyre char blend (OTC). Chars were produced through slow pyrolysis at 550 °C under an inert nitrogen atmosphere. At two low filler loadings (1 and 2 wt.%), we compare composites produced via solvent casting and melt mixing to elucidate how preparation routes modulate dispersion quality and interfacial behavior. Comprehensive physicochemical, thermal, electrical, and mechanical characterizations were conducted to establish correlations between processing, structure, and properties. Through this systematic evaluation, we aim to identify optimal formulations and processing conditions that maximize material performance and expand the potential applications of PLA across multiple sectors: electronics, automotive applications (e.g., antistatic components and lightweight structural materials), and energy-related devices.

## 2. Materials and Methods

### 2.1. Materials and Char Preparation

In this study, PLA pellets supplied by NatureWorks^®^ (Plymouth, MN, USA) were used as the polymer matrix. They contain approximately 4.3 wt.% D-lactide and 95.7 wt.% L-lactide [16], and are therefore classified as PLLA. Chloroform was employed for dissolving PLLA (CHCl_3_, PanReac AppliChem^®^, Darmstadt, Germany). Three types of char were produced through slow pyrolysis at 550 °C in an inert nitrogen atmosphere. The first, designated as OC (Olive Char), was obtained from olive tree pruning waste; the second, TC (Tyre Char), was derived from end-of-life tyres; the third, OTC, was a 1:1 (wt./wt.) mixture of the two previous chars, co-pyrolyzed under the same conditions. Pyrolysis was carried out in a tubular furnace (Carbolite TZF 12/65/550, Sheffield, UK) under a nitrogen atmosphere maintained at 4 mL/min to ensure oxygen-free conditions. The temperature was increased at a rate of 10 °C/min up to 550 °C, where it was held isothermally for 30 min to complete carbonization. Afterward, the system was allowed to cool naturally to room temperature while the nitrogen flow continued, preventing oxidation of the resulting carbonaceous product. Before preparing the composites, both PLLA pellets and the produced char powders were dried overnight at 60 °C to remove residual moisture. The char powders were further dried at 100 °C for 2 h to ensure the complete removal of adsorbed water.

### 2.2. Characterization of Chars

FTIR spectra were recorded using a Jasco FT/IR-6100 spectrometer (Tokio, Japan) (0.5 cm^−1^ resolution, range 4000–400 cm^−1^) with KBr pellets prepared in a 50:1 ratio for OC and OTC, and 75:1 for TC. Raman spectroscopy was performed using a Renishaw^®^ InVia Raman microscope (Wotton-under-Edge, UK) equipped with a 532 nm laser to assess carbon structure through D and G band analysis. Morphological and microstructural features were examined via a ZEISS EVO 50 XVP SEM (Oberkochen, Germany) (15 kV), with EDX employed for elemental composition analysis.

### 2.3. Preparation of PLLA/Char Composites

Two different fabrication methods were investigated to optimize filler dispersion in the PLLA matrix: solvent casting and melt mixing.

#### 2.3.1. Solvent Casting Method

PLLA was dissolved in chloroform (5 wt./vol.%) under continuous stirring at room temperature. The desired amount of char (1 and 2 wt.%) was added and stirred for 4 h. Approximately 35 g of the resulting solution was poured into nylon-coated Petri dishes (80 mm diameter) and dried overnight to obtain films of ~0.3 mm thickness. Herein, the samples obtained by solvent casting are designated as (PLLA/Cw)*_c_* where C denotes the type of char (i.e., OC, TC, OTC) and w represents the char content in the formulation (i.e., 1 or 2 wt.%). The subscript *c* out of the round brackets indicates the solvent casting process. Images of the casted films are reported in the Supplementary Materials (Figure S1).

#### 2.3.2. Melt Mixing Method

PLLA pellets and char were blended at 170 °C using a Brabender W50E plastograph (Duisburg, Germany) at 100 rpm for 5 min. Composite blends containing 1 and 2 wt.% filler were then crushed and hot pressed using a Collin P200T laboratory press (Maitenbeth, Germany). Pressing was performed at 170 °C for 5 min under 100 bar. Either square (70 mm× 70 mm, 0.5 mm thick) or circular (80 mm diameter, 0.5 mm thick) samples were prepared to undergo different characterizations. Herein, the samples obtained by melt mixing are designated as (PLLA/Cw)*_m_* where C denotes the type of char (i.e., OC, TC, OTC) and w represents the char content in the formulation (1 or 2 wt.%). The *m* out of the round brackets indicates the melt mixing process. Images of these films are reported in the Supplementary Materials (Figure S2).

### 2.4. Characterization of PLLA Composites

#### 2.4.1. Morphological and Structural Analysis

Morphological and microstructural features were examined via a ZEISS EVO 50 XVP SEM (15 kV) (Oberkochen, Germany). X-ray diffraction (XRD) analysis was conducted using a PANalytical X’Pert PRO MPD (Almelo, The Netherlands) diffractometer with CuKα radiation (λ = 0.15418 nm), operating at 45 kV and 40 mA in Bragg-Brentano geometry. The degree of crystallinity ($\chi_{C}$ (WAXS)) was calculated using the ratio between the area under the crystalline peaks (*A*_C_) and the total area, including the amorphous contribution (*A*_A_), as follows:

(1) $$\chi_{C}(WAXS)=\frac{A_{c}}{A_{c}-A_{A}}\cdot 100$$

FTIR spectra were recorded using a Jasco FT/IR-6100 (Tokio, Japan) spectrometer (0.5 cm^−1^ resolution, 4000–400 cm^−1^ range) in ATR mode.

#### 2.4.2. Thermal Analysis

Differential Scanning Calorimetry (DSC) analysis was carried out using a DSC Q2000 (TA Instruments, New Castle, DE, USA) on samples weighing approximately 7 mg. Each sample underwent a thermal cycle consisting of heating from 0 °C to 200 °C at a rate of 10 °C/min, followed by an isothermal hold at 200 °C for 3 min, cooling to 0 °C at 10 °C/min, and a second heating to 200 °C at the same rate. The degree of crystallinity ($X_{c}$ (DSC)) was calculated using the Equation (2):

(2) $$X_{c}(DSC)=\frac{\Delta H_{f}}{\Delta H_{f}^{0}\;W_{PLLA}}\cdot 100$$

where $\Delta H_{f}$ is the measured melting enthalpy, $\Delta H_{f}^{0}$ = 93.2 J/g is the theoretical melting enthalpy of 100% crystalline PLLA, and *W*_PLLA_ is the weight fraction of PLLA in the composite.

#### 2.4.3. Mechanical Properties

For mechanical characterization, uniaxial tensile tests were performed using an Instron 5966 dynamometer (Norwood, MA, USA) equipped with a 2 kN load cell, following the ASTM D638 standard [17]. Rectangular specimens (40 × 5 × 1 mm^3^) of thickness equal to 1 mm, were tested at a constant rate of 2 mm/min. The main parameters evaluated were Young’s modulus (*E*), elongation at yield (ε_y_), strength at yield (σ_y_), elongation at break (ε_b_) and strength at break (σ_y_), allowing comparison of stiffness, strength, and ductility across different composite formulations.

#### 2.4.4. Electrical Properties

The electrical measurements were performed using a high-input-impedance electrometer (Keithley 6517B, Cleveland, OH, USA) connected to a guarded resistivity test fixture (Model 8009, Cleveland, OH, USA), specifically designed for evaluating the volume resistivity of flat insulating samples.

## 3. Results and Discussion

### 3.1. Char Characterization

SEM analysis shows that the three chars have distinct morphologies linked to their origins. OC (Figure S3A,B) has a highly porous, fibrous structure with microchannels, which can enhance polymer infiltration and mechanical bonding with PLLA [18]. In contrast, TC (Figure S3C,D) show dense, granular particles with irregular and compact morphologies, ranging from tens to hundreds of micrometers. The hybrid OTC combines both features, i.e., OC’s macroporosity and TC’s nanostructured particles (Figure S3E,F).

EDX analysis (Figure S4) reveals distinct compositional features among the three chars. The olive-derived biochar (OC) displays a clean C–O profile, typical of lignocellulosic biomass pyrolyzed at moderate temperatures, confirming its high purity [19]. In contrast, the tyre char (TC) is dominated by carbon and shows no oxygen peak, with only a very small sulphur signal arising from sulphur-based vulcanization additives. Literature reports that tyre chars commonly contain more complex inorganic components such as Ca, Ti, Zn, S, and Mo originating from the employed additives and vulcanization agents [20,21]; however, these elements are not detected in the spectra. Their absence is attributed to the limited sensitivity of the EDX instrument used in this work, as low-concentration inorganic constituents may fall below the detection threshold (i.e., at 1%). The hybrid OTC sample exhibits a carbon-rich spectrum like TC but retains a small oxygen contribution from the OC fraction, along with a very weak Si peak likely associated with minor inorganic residues.

The FTIR spectrum of OC (Figure S5) exhibits broad O–H and N–H stretching vibrations (associated with phenolic and amine functionalities) in the 3500–3000 cm^−1^ region, along with C–H symmetric stretching bands typical of lignocellulosic materials between 3000 and 2800 cm^−1^. More detailed structural information is obtained from the fingerprint region (1800–400 cm^−1^). The CH_2_ wagging vibration at 1312 cm^−1^ indicates the presence of cellulose and hemicellulose (in salt or ester forms) [22]. Conversely, other characteristic cellulose-related bands at 1031 cm^−1^ (C–O stretching of cellulose and its derivatives) [23], 1371 cm^−1^ (aliphatic CH_3_ deformation), and 1243 cm^−1^ (C–O stretching of cellulose and hemicellulose) are absent, likely due to the pyrolysis temperature exceeding the cellulose devolatilization threshold [24]. The band at 1604 cm^−1^ is attributed to aromatic skeletal vibrations of lignin [25] and to C = O stretching contributions from lignin and residual hemicellulose [26].

The FTIR spectrum (Figure S5) of the TC is dominated by bands associated with residual saturated hydrocarbons, evidenced by the CH_3_ bending vibration at 1385 cm^−1^, and with condensed aromatic and polyaromatic structures, as indicated by the C = C stretching band at 1609 cm^−1^. A weak band close to 2900 cm^−1^ corresponds to aliphatic C–H stretching, suggesting the presence of incompletely pyrolyzed rubber residues. The absence of O–H and N–H stretching bands indicates a more hydrophobic surface and a higher degree of carbonization, characteristics commonly observed in rubber-derived or industrial carbonaceous materials [27].

Raman analysis (Figure S6) confirms the presence of the typical D (~1360 cm^−1^) and G bands (~1590 cm^−1^) in all samples, enabling comparison of structural order through the I_D_/I_G_ ratio [28,29]. TC shows the highest ratio (~1.8), reflecting a highly disordered, amorphous carbon structure produced by the pyrolysis of rubber and tyre additives. OC displays a moderately lower ratio (~1.5), consistent with the slightly more ordered aromatic carbon typical of biomass-derived chars [30]. The hybrid char (OTC) exhibits the lowest I_D_/I_G_ ratio (~1.1), indicating a higher degree of structural order compared to both TC and OC. This behavior can be attributed to synergistic effects occurring during the co-pyrolysis of tyre rubber and olive biomass. The combination of their respective volatile species and mineral contents promotes more efficient carbon reorganization: tyre-derived aromatic fragments facilitate graphitic layer growth, while biomass-derived oxygenated intermediates enhance molecular mobility and assist the rearrangement of highly crosslinked tyre residues. Additionally, the mixed inorganic fraction comprising Zn-, Si-, Ca-, K-, and Mg-based species is a more effective catalyst for graphitization than either precursor alone. Together, these effects reduce defect density and support the formation of larger, better-ordered aromatic domains, thereby explaining the reduced I_D_/I_G_ ratio observed for OTC.

### 3.2. Morphological, Structural, and Thermal Characterization of Composites

**Figure 1:**
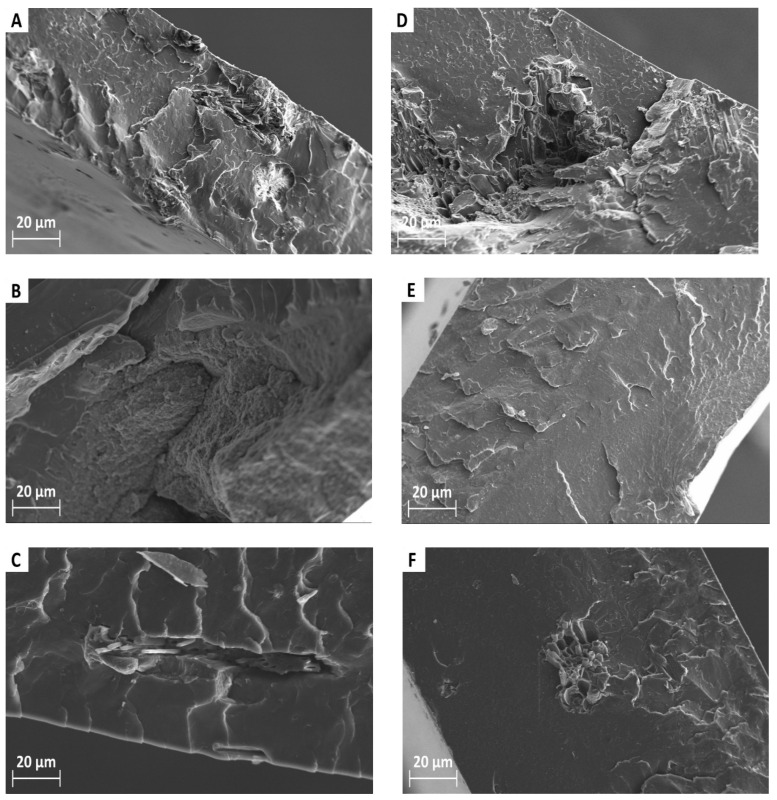
Scanning Electron Microscopy (SEM) images of samples incorporating 2 wt.% fillers, acquired in secondary electron (SE) mode, prepared by (**A**–**C**) solvent casting and (**D**–**F**) melt mixing: (**A**,**D**) OC, (**B**,**E**) TC, and (**C**,**F**) OTC fillers.

Figure 1A–C reports the typical SEM cross sectional micrographs of PLLA composites prepared via solvent casting and melt mixing. Figure 1A, corresponding to the (PLLA/OC2)*_c_* sample, shows large OC particles dispersed in the PLLA matrix with voids and poorly bonded regions throughout the cross-section [31]. These features highlight the limitations of solvent casting at 2 wt.% filler loading, where sedimentation and insufficient wetting hinder uniform integration of char within the polymer matrix [2].

Also, when TC is used as a filler, it is clearly not uniformly dispersed within the PLLA matrix, with large agglomerates evident, some of which approach or exceed 100 µm (Figure 1B, sample (PLLA/TC1)*_c_*). This aggregation suggests a strong tendency of tyre-derived char to form clumps during solvent evaporation, likely due to its fine particle size and low surface polarity, which reduce its compatibility with the PLLA matrix [32]. Increasing the filler content further exacerbates this issue, leading to larger and more frequent particle clusters. Similar trends were reported for mineral fillers produced by mechanical crushing and grinding and incorporated into cementitious matrices [31], as well as for hydrophobic phytochemical compounds dispersed in aqueous media via magnetic stirring–assisted extraction [2], where limited matrix–filler affinity and increasing concentration promote particle clustering.

One of the char particles in which PLLA is incorporated is shown in Figure 1C ((PLLA/OTC1)*_c_* sample). The observed microstructure highlights the limitations of solvent casting for achieving uniform filler distribution, particularly in hybrid composites and especially when working with finely divided and hydrophobic fillers like carbonized rubber. Figure 1D–F shows the SEM micrographs of PLLA composites obtained via melt mixing. Figure 1D shows that polymer filaments extend from the porous channels of the (PLLA/OC1)*_m_* sample. Compared to casting, melt mixing enhances filler embedding within the matrix and reduces the presence of large voids. These findings are consistent with those reported in the literature [6], where PLA-based composites filled with biochar derived from spent ground coffee and prepared by melt mixing and solvent casting exhibited similar behaviour. [9]. The filler appears more uniformly dispersed, especially at 1 wt.%, although some heterogeneity persists at 2 wt.% due to particle size variability. No significant fragmentation or surface damage is observed, suggesting that melt mixing preserves the structural integrity of the filler [33]. In Figure 1E ((PLLA/TC1)*_m_* sample), the filler appears more uniformly distributed, with significantly fewer and smaller aggregates. Both 1 and 2 wt.% samples show improved dispersion, suggesting that the high shear stresses applied during melt mixing effectively limit agglomeration [34]. The same morphology is observed for (PLLA/OTC1)*_m_* (Figure 1F) with well-dispersed filler compared to solvent casting. Indeed, melt mixing produces more compact and uniform morphology, resulting in improved filler–matrix interaction and reduced agglomeration.

Depending on the preparation method used, PLLA can crystallize into α, α′, β, or γ forms [35].

**Figure 2:**
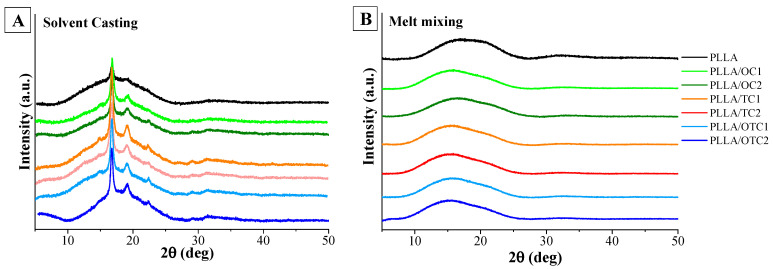
Wide Angle X-ray Diffraction (WAXD) patterns of PLLA and PLLA-based composites obtained by (**A**) solvent casting and (**B**) melt mixing.

Figure 2A presents the diffraction patterns of PLLA-based composite films, prepared by solvent casting, and recorded in the 2θ range from 5° to 50°.

All samples display a prominent diffraction peak around 16.6°, corresponding to the 110/200 reflections of α form of semi-crystalline PLLA [36]. As illustrated in the patterns, the addition of char, regardless of type or loading, does not noticeably shift the primary diffraction peak at 16.6°, suggesting that the crystalline structure of PLLA remains largely unaffected. The crystallinity index (χ_C_), calculated using Equation (1) and reported in Table 1, shows no appreciable variation among the composites prepared with different chars or at different loadings. In all cases, the crystallinity of the composites exceeds that of unfilled PLLA (i.e., 12%).

When prepared by melt mixing (Figure 2B), PLLA exhibits an amorphous halo centered at 2θ ≈ 16.6°, indicating that the rapid cooling of the melt prevents crystallization of PLLA into the α form [37]. The PLLA broad scattering halo is characterized by the superposition of two halos picked at 2θ values of ~16.5° and ~20.4°. This scattering behavior closely resembles that of PET, as reported by Murthy et al. [38] and has also been observed in PLLA. Such pattern suggests the existence of two distinct interchain spacings, as previously described by Stoclet et al. [39]. The diffractograms of all the composites show two main peaks, as in unfilled PLLA, the first of which, however, is shifted to lower 2θ values and presents a higher relative intensity with respect to the second one. To check if these changes in the diffractograms are due to the partial crystallization of PLLA in the mesomorphic form [40], FTIR spectroscopy in ATR mode was performed.

**Figure 3:**
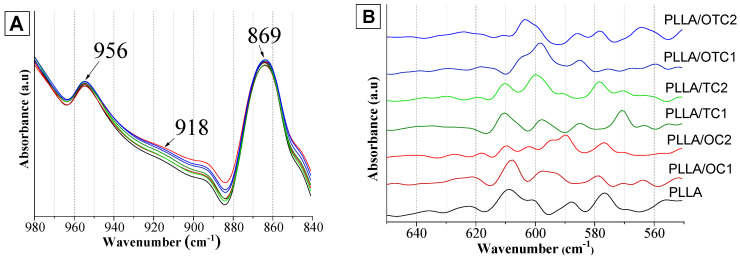
FTIR/ATR spectra of PLLA-based composites (**A**) in the range 980–840 cm^−1^ region and (**B**) in the low-frequency region.

Figure 3A presents the FTIR/ATR spectra of PLLA and its melt-mixed composites in the 980–840 cm^−1^ region. The *v*(C–COO) band at 869 cm^−1^ remains invariant for both unfilled PLLA and its composites, demonstrating that the incorporation of char fillers does not modify the structural ordering of the polymer matrix [41]. In addition, the band at 918 cm^−1^ typically assigned to the coupled *v*(C–COO) and *r*(CH_3_) vibrations and considered as a spectroscopic marker of the PLLA mesophase [40] is not observed in any of the spectra shown in Figure 3A. Conversely, all samples exhibit a band at 956 cm^−1^, which is associated with the conventional amorphous state of PLLA [41]; its intensity and position are comparable to those of unfilled PLLA.

These observations indicate that the differences detected in the WAXD patterns cannot be attributed to mesophase formation. At variance, they more likely arise from the occurrence of specific interactions between the fillers and the polymer matrix, which perturb the local packing of the amorphous phase. This interpretation is supported by the analysis of the low-frequency region of the FTIR/ATR spectra (Figure 3B). In the 650–550 cm^−1^ range, the CH_3_ rocking and backbone bending vibrations exhibit pronounced changes in both intensity and peak position as a function of char content and nature, demonstrating that the fillers significantly affect chain mobility and the amorphous packing environment.

**Figure 4:**
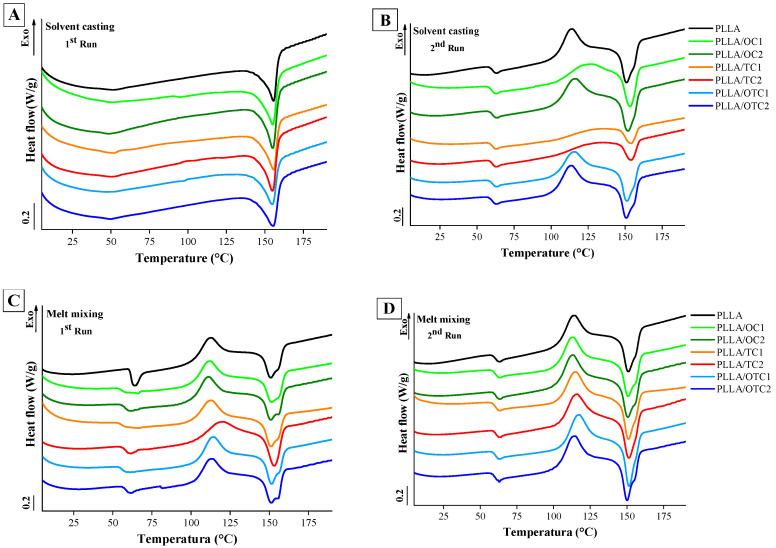
Traces of samples obtained by (**A,B**) solvent casting and (**C,D**) melt mixing, including both first and second heating scans.

Differential Scanning Calorimetry (DSC) was used to assess the thermal behavior of PLLA-based composites filled with different chars and processed by solvent casting and melt mixing. Figure 4 presents the curves from both the first and second heating cycles. Cooling runs are reported in the Supplementary Information (Figure S7). Table 2 summarizes the thermal parameters and the degrees of crystallinity ($X_{c}$%) calculated by Equation (2).

For the samples obtained by solvent casting, in the first heating run (Figure 4A), a single sharp melting peak (*T_m_*_1_) around 156 °C is visible, corresponding to the melting of crystalline PLLA domains. No significant differences are observed between unfilled PLLA and its composites at this stage. As shown in Table 2, the degree of crystallinity ($X_{c}$%) of unfilled PLLA is 15%, while composite films exhibit values between 21% (PLLA/TC1)*_c_* and 23% (PLLA/OTC1)*_c_* confirming the WAXD results.

During the second heating (Figure 4B), a clear glass transition (*T_g_*) is observed around 60 °C for pure (PLLA)*_c_*. The *T_g_* values of all composite films remain within the close range of 60–61 °C, showing that the addition of char does not significantly affect the mobility of the PLLA amorphous phase, regardless of filler type or loading [42].

Following *T_g_*, a distinct cold crystallization peak (*T_c_*) appears near 120 °C for PLLA and around 113–122 °C for its composites. This exothermic event corresponds to the reorganization of amorphous chains into crystalline regions, which occurs upon heating due to the insufficient time for crystallization during the cooling run.

In the melting region, two peaks are visible (*T_m_*_1_ and *T_m_*_2_) for almost all the composites. This is widely reported in the literature and is ascribed to the lamellar reorganization and crystals size [43,44] with the higher temperature peak related to more highly organized crystals [43]. Figure 4C,D illustrate the curves of the samples obtained via melt mixing: the thermal parameters are collected in Table 2. In the first heating scan (Figure 4C), the glass transition (*T_g_*), at ≈ 60°, is followed by an endothermic peak due to relaxation phenomena of amorphous chains [45]. The relaxation peak is especially pronounced for the unfilled PLLA samples. Cold crystallization (*T_c_*) is observed already in the first run, between 110 °C and 118 °C, confirming the prevalently amorphous nature of the samples obtained by melt mixing. In the melting region, two peaks are visible (*T_m1_* and *T_m2_*) at about 151 °C and 155 °C: again, no influence of the char is observed. The crystallinity, calculated by subtracting the crystallisation enthalpy ($\Delta H_{c})$ of the first run from the melting enthalpy ($\Delta H_{f})$, and calculated using Equation (2), is always below 2%, which confirms the results of WAXD analysis.

In the second heating scan (Figure 4D), *T_c_*, *T_m_*_1_ and *T_m_*_2_ are not significantly affected by the presence of char. Conversely, the *T*_g_ values increase by increasing the char amount. This confirms that due to the mixing process, the char influences more the mobility of the PLLA chains.

**Figure 5:**
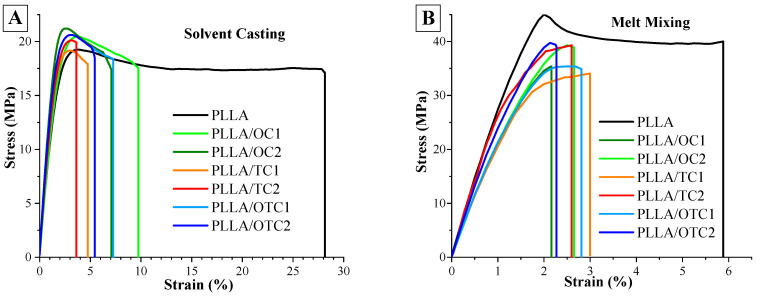
Stress-strain curves obtained in tensile configuration for PLLA composites obtained by (**A**) solvent casting and (**B**) melt mixing.

**Figure 6:**
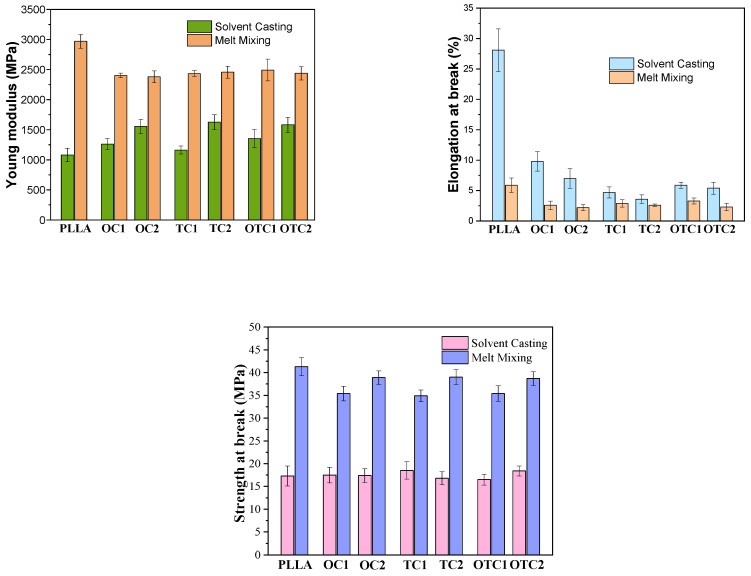
Comparison of Young’s Modulus, elongation at break and strength at break parameters among composites prepared by solvent casting and melt mixing.

The mechanical properties of the PLLA and composites prepared by solvent casting and melt mixing are summarized in Figure 5 and Figure 6. (PLLA)_c_ demonstrates a pronounced ductile mechanical response, characterized by a Young’s modulus of 1082 MPa, a tensile strength of 17 MPa, and an elongation at break of 28%. The onset of plastic deformation is observed at a yield strain (ɛᵧ) of about 4% and a corresponding yield stress (σᵧ) of 19 MPa.

By contrast, (PLLA)_m_ displays a substantially higher Young’s modulus (2970 MPa), tensile strength (38 MPa), and yield stress (45 MPa), along with a pronounced reduction in elongation at yield (2%) and at break (6%). Compared to (PLLA)_c_, this corresponds to an approximately threefold increase in stiffness and a twofold increase in strength, accompanied by a fourfold decrease in ductility, which can be ascribed to differences in processing routes. During solvent casting, polymer chains solidify under minimal external deformation, leading to a more relaxed and spatially extended arrangement with a lower entanglement density. Conversely, melt mixing imposes substantial shear and stretching, promoting tighter chain packing and a higher degree of entanglement [46].

As a result, the solvent-cast material exhibits greater chain mobility and allows segments to slide past one another more readily, yielding lower strength and stiffness but enhanced ductility.

The addition of char leads to a substantial reduction in ductility across all methods. When char is incorporated into solvent-cast PLLA, the Young’s modulus increases, whereas the tensile strength remains essentially unchanged across the composite series (Figure 6). All solvent-cast composites exhibit a distinct yield point that remains mainly unaffected by the presence of char. In contrast, the addition of char to melt-mixed PLLA results in a reduction in both modulus and strength (Figure 6) and leads to the disappearance of the yielding point. These different trends can be rationalized by considering (i) the different polymer microstructures generated by the two processing methods and (ii) the intrinsic characteristics of the various chars. In solvent casted PLLA, the polymer chains are less entangled and more loosely packed; therefore, the introduction of rigid char particles can act as an effective reinforcing phase, restricting local chain mobility and increasing stiffness without significantly disrupting load-bearing continuity.

In melt-mixed PLLA, however, the polymer exhibits a higher entanglement density and a more compact amorphous structure. In this case, the addition of the char, as demonstrated by WAXD and FTIR, interferes with the chain packing, creating interfacial defects and/or reducing the effective stress-transfer capability.

Table 3 reports the electrical resistivity values of the PLLA-based composites produced using the two processing methods. The unfilled (PLLA)*_c_* sample exhibits a very high resistivity of 123 × 10^15^ Ω·cm, confirming its strongly insulating character, consistent with previous findings for pure PLA matrices [47]. Adding char fillers generally decreases resistivity with increasing content, although the values remain within the same order of magnitude of unfilled PLLA. The lowest resistivity among the solvent-cast samples is observed for (PLLA/OTC2)*_c_* (i.e., 4 × 10^15^ Ω·cm).

(PLLA)*_m_* shows a resistivity of 20 × 10^15^ Ω·cm. For the melt-mixed composites, the incorporation of olive biochar significantly reduces volume resistivity, whereas the (PLLA/TC)*_m_* composite shows resistivity values similar to or even higher than unfilled PLLA (41 × 10^15^ Ω·cm for (PLLA/TC1)*_m_* and 21 × 10^15^ Ω·cm for (PLLA/TC2)*_m_*). The hybrid OTC filler yields the most pronounced resistivity reduction, particularly at 2 wt.% loading, where (PLLA/OTC2)*_m_* reaches 0.02 × 10^15^ Ω·cm. These results indicate that melt mixing combined with an optimized hybrid char composition improves electrical transport, although the materials remain insulating overall [48,49]. The superior performance of the OTC hybrid char is likely due to complementary carbon structures generated from the two feedstocks during pyrolysis: tyre waste provides conductive graphitic domains, while olive residues account for porosity and enhance packing. Together, these features promote a more continuous and better-connected “conductive network” than either char alone.

## 4. Conclusions

This study demonstrates that the combination of waste-derived chars and processing strategies is crucial for tailoring the performance of PLLA composites while advancing circular-economy goals. Chars from olive pruning waste (OC), end-of-life tyres (TC), and their co-pyrolyzed hybrid (OTC) exhibited distinct carbon structures, with I_D_/I_G_ ratios of 1.5, 1.8, and 1.1, respectively, confirming superior structural order for the hybrid filler. Processing played a dominant role: solvent casting produced micrometric agglomerates (>100 µm), whereas melt mixing ensured homogeneous dispersion and improved filler-matrix embedding, with crystallinity values of 20–25% for cast samples and below 2% for melt-mixed counterparts. Thermal properties remained largely unchanged although melt-mixed samples exhibited slight increase in *T_g_* of a few degrees, corresponding to restricted chain mobility due to the presence of chars. The mechanical behavior reflected differences in polymer microstructure, with unfilled PLLA showing E ≈ 1082 MPa (cast) versus 2970 MPa (melt-mixed), and all composites exhibiting reduced ductility. The most pronounced enhancement concerned electrical resistivity, where melt-mixed OTC at 2 wt.% loading achieved 0.02 × 10^15^ Ω·cm, a twofold decrease with respect to unfilled PLA. From a general point of view, PLLA/char composites are important from a socio-economic perspective because they combine biodegradability with enhanced functional properties, offering a sustainable alternative to conventional plastics. Their use can reduce environmental pollution, lower reliance on fossil-fuel-based materials, and promote value-added utilization of waste mass and biomass, supporting both circular economy initiatives and green manufacturing industries. The developed PLLA/char composites are promising candidates for applications in flexible electronics, sensors, and antistatic components, as well as in lightweight structural materials and energy devices. Moreover, further improvements in PLLA/char composites can be achieved by enhancing the interfacial adhesion through surface modification of the char, such as chemical functionalization or silanization. Additionally, the incorporation of a third component, such as conductive nanofillers or rigid nanoparticles, can provide synergistic effects and tailor the overall properties.

## Figures and Tables

**Table 1:** Degree of crystallinity ($\chi_{C}$) by WAXS of PLLA composites prepared by solvent casting.

Sample	$\chi_{C}$ (%)
PLLA	12 ± 1
(PLLA/OC1)*_c_*	20 ± 1
(PLLA/TC1)*_c_*	21 ± 1
(PLLA/OTC1)*_c_*	21 ± 1
(PLLA/OC2)*_c_*	22 ± 1
(PLLA/TC2)*_c_*	20 ± 1
(PLLA/OTC2)*_c_*	20 ± 1

**Table 2:** Thermal parameters of PLLA-based samples prepared by solvent casting and melt mixing, including glass transition temperature (*T_g_*), crystallization temperature (*T_c_*), and melting temperatures (*Tm*_1_*, Tm*_2_) obtained during the first and second heating runs, along with the degree of crystallinity ($X_{c}$).

		1st Run					2nd Run			
		*T_g_* (°C)	*T_c_* (°C)	*T_m_*_1_ (°C)	*T_m_*_2_ (°C)	$X_{c}$ (%)	*T_g_* (°C)	*T_c_* (°C)	*T_m_*_1_ (°C)	*T_m_*_2_ (°C)
Solvent casting	PLLA	-	-	156	-	15.0	60	120	153	158
	PLLA/OC1	-	-	155	-	22.2	60	122	153	-
	PLLA/OC2	-	-	155	-	22.9	60	115	152	-
	PLLA/TC1	-	-	156	-	19.6	61	-	154	-
	PLLA/TC2	-	-	156	-	22.9	60	-	154	-
	PLLA/OTC1	-	-	155	-	24.7	61	115	151	158
	PLLA/OTC2	-	-	155	-	24.2	60	113	151	158
Melt mixing	PLLA	60	113	151	158	1.4	58	112	151	158
	PLLA/OC1	61	112	151	158	1.6	60	112	152	158
	PLLA/OC2	61	110	151	158	0.4	60	112	151	157
	PLLA/TC1	61	114	151	158	4.1	59	112	151	158
	PLLA/TC2	60	118	153	-	2.3	61	114	151	-
	PLLA/OTC1	61	117	152	158	0.3	59	114	152	158
	PLLA/OTC2	61	112	151	158	2.0	61	113	150	157

**Table 3:** Comparison of Electrical Resistivity Values Among Preparation Methods.

Resistivity (Ω·cm)·10^15^						
	Solvent Casting			Melt Mixing		
Char	OC	TC	OTC	OC	TC	OTC
0 wt. %	123			20		
1 wt. %	21	110	17	0.1	41	0.2
2 wt. %	5	97	4	0.08	21	0.02

## Data Availability

The original contributions presented in this study are included in the article/Supplementary Materials. Further inquiries can be directed to the corresponding authors.

## Supplementary Materials

The following supporting information can be downloaded at: https://www.mdpi.com/article/10.3390/polym18070871/s1, Figure S1: PLLA films produced by solvent casting with (A) 1 wt% and (B) 2 wt% filler content. From left to right: OC, TC, and OTC; Figure S2: PLLA films produced by melt mixing with (A) 1 wt% and (B) 2 wt% filler content. From left to right: OC, TC, and OTC; Figure S3: SEM micrographs of A, B) OC, C, D) TC and E, F) OTC fillers; Figure S4: EDX analysis of the OC, TC, and OTC fillers; Figure S5: FTIR spectra of the OC, TC, and OTC fillers; Figure S6: Raman spectra of the OC, TC, and OTC fillers; Figure S7: Cooling runs of PLLA samples obtained by (A) solvent casting and (B) melt mixing.
