# Supplementary material for: Impact of Processing and Char Feedstock on the Thermal, Mechanical, and Electrical Behavior of PLLA Composites
Source: Polymers (Basel). 2026 Apr 1;18(7):871. doi: 10.3390/polym18070871 (PMC13075034; doi:10.3390/polym18070871)
Supplement: Supplementary file 1 [file polymers-18-00871-s001.zip › polymers-4224087-supplementary.pdf]

Supplementary material

Impact of Processing and Char Feedstock on the Thermal, Mechanical, and Electrical Behavior of PLLA Composites

Donatella Duraccio[a*], Boutheina Rzig[a*], Mattia Di Maro[a], Giulio Malucelli[b], Finizia Auriemma[c], Federica Pignatelli[d], Giuliana Magnacca[d], Pier Paolo Capra[e], Mattia Bartoli[f,g], Maria Giulia Faga[a]

[a] *Istituto di Scienze e Tecnologie per l'Energia e la Mobilità Sostenibili, Consiglio Nazionale delle Ricerche, Strada delle cacce, 73-1035 Torino, Italy*

[b] *Dipartimento di Scienza Applicata e Tecnologia, Politecnico di Torino, Corso Duca degli Abruzzi 24, 10123 Torino, Italy*

[c] *Dipartimento di Chimica "Paolo Corradini", Università di Napoli "Federico II", Complesso Monte S. Angelo, Via Cintia, I-80126 Napoli, Italy*

[d] *Dipartimento di Chimica, Università di Torino, Via P. Giuria 7, 10125 Torino, Italy*

[e] *Istituto Nazionale di Ricerca Metrologica, Strada delle cacce, 91-1035 Torino, Italy*

[f] *Istituto Italiano di tecnologia- Center for Sustainable Future Technologies, Via Livorno 60, 10144 Torino, Italy*

[g] *Consorzio Interuniversitario Nazionale per la Scienza e Tecnologia dei Materiali (INSTM), Via G. Giusti 9, 50121 Florence, Italy*

*Corresponding authors:

Donatella Duraccio

Email: donatella.duraccio@stems.cnr.it

Tél: +39 011 39 77 741

Boutheina Rzig

Email: boutheina.rzig@stems.cnr.it

Tél: +393520412376

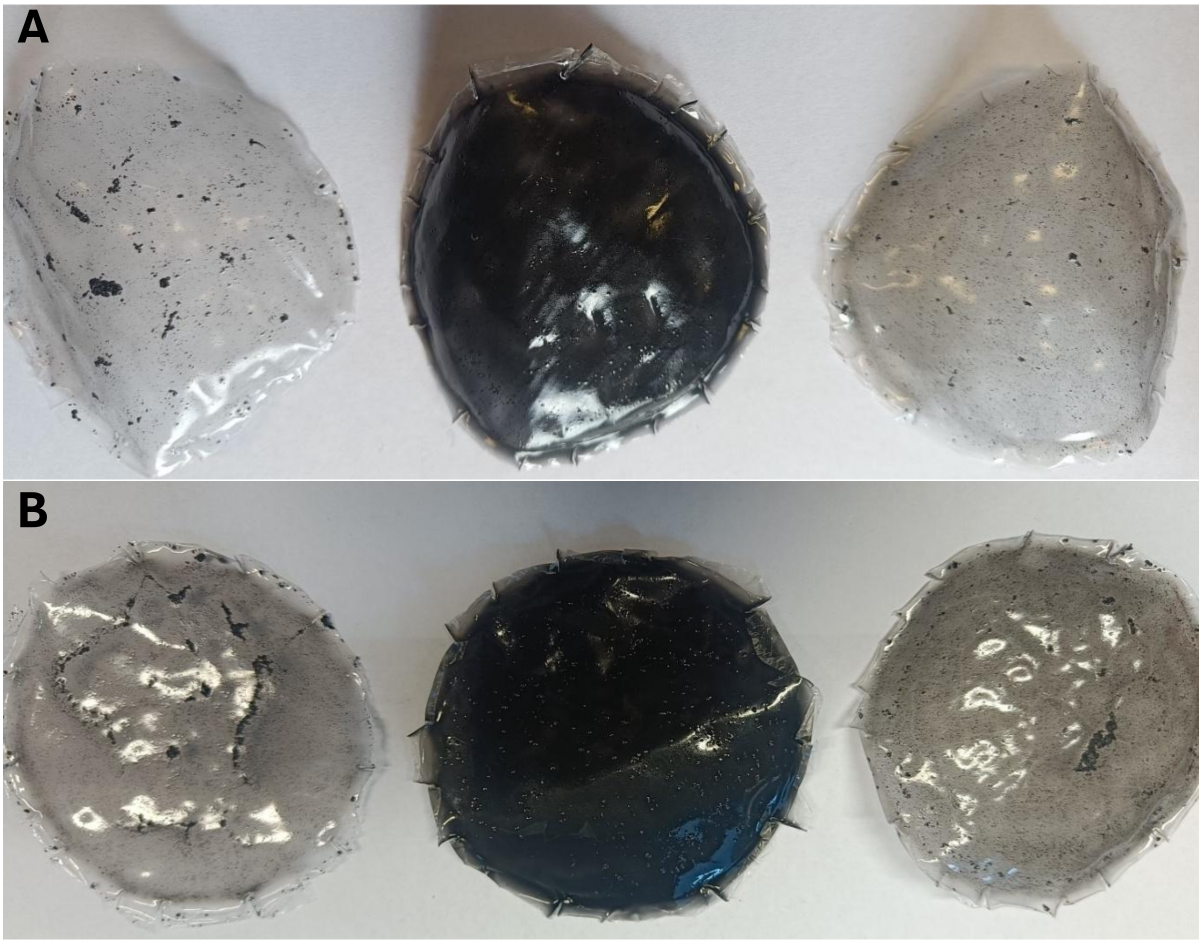

Figure S1. PLLA films produced by solvent casting with (A) 1 wt% and (B) 2 wt% filler content. From left to right: OC, TC, and OTC.

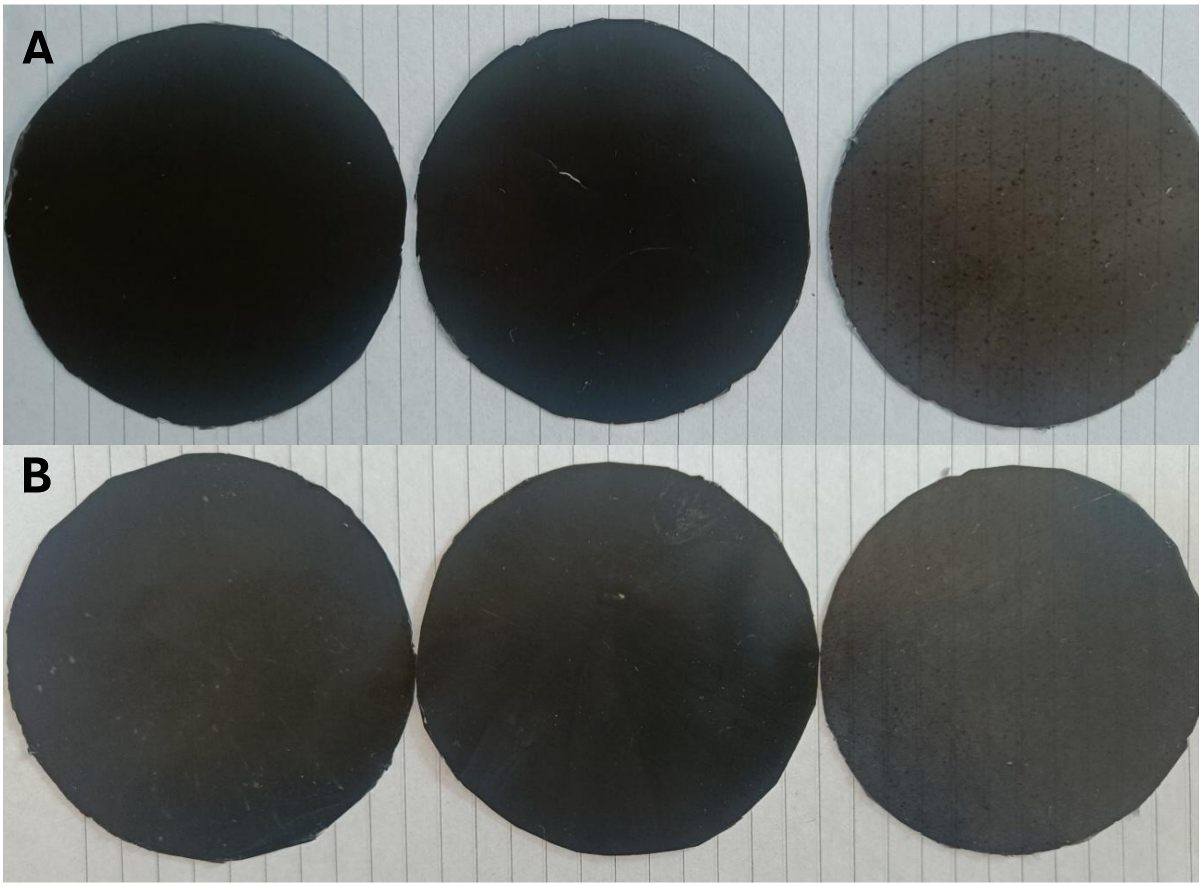

Figure S2. PLLA films produced by melt mixing with (A) 1 wt% and (B) 2 wt% filler content. From left to right: OC, TC, and OTC.

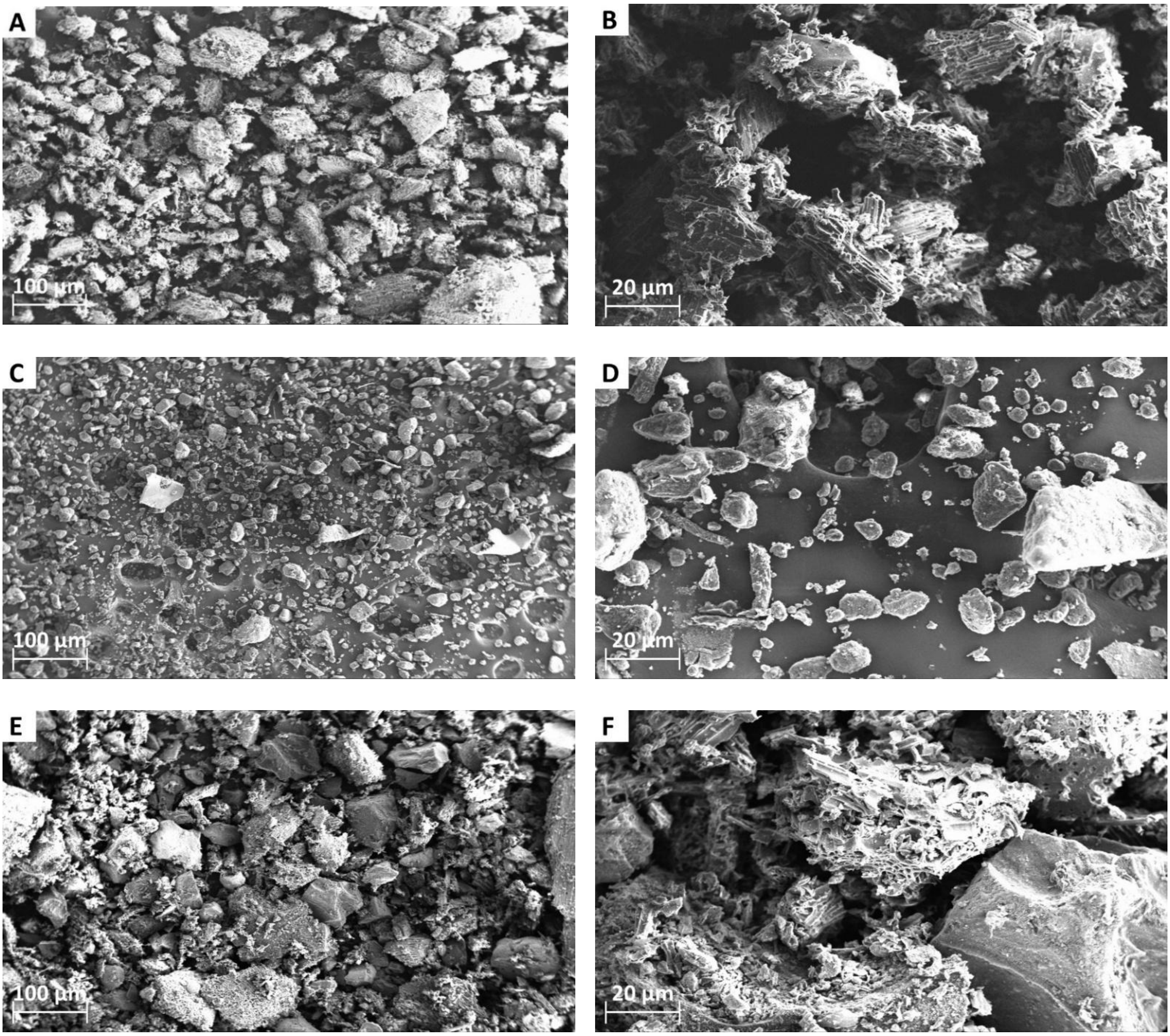

Figure S3. SEM micrographs of **A, B)** OC, **C, D)** TC and **E, F)** OTC fillers.

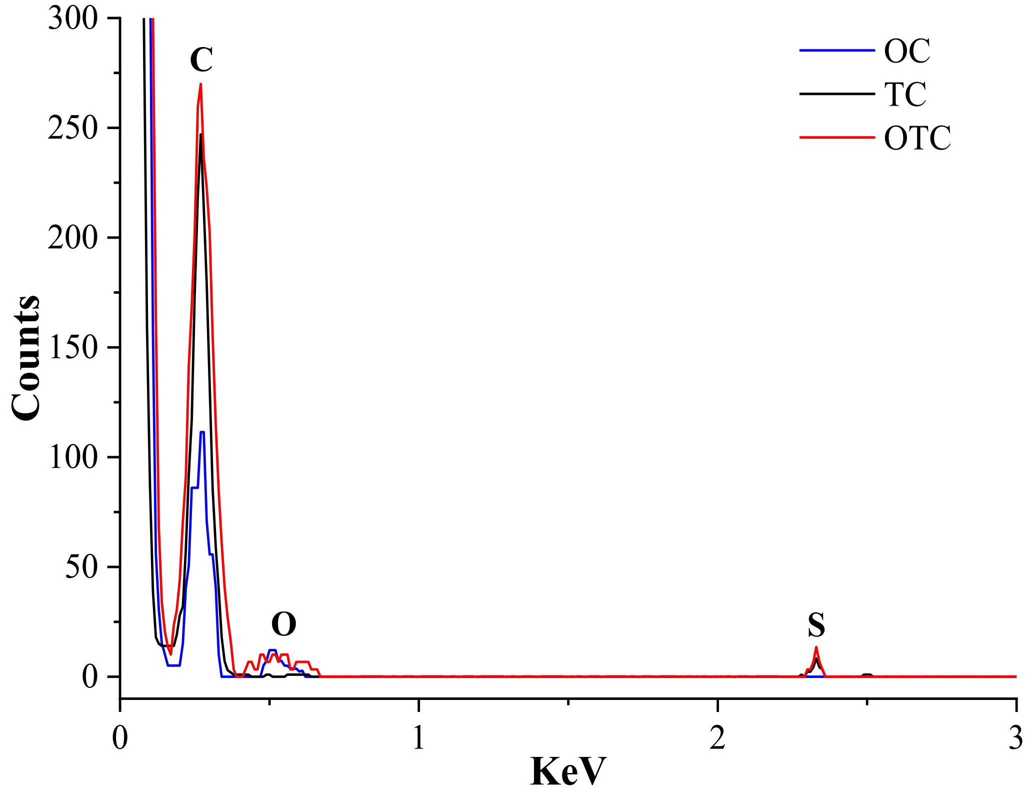

Figure S4. EDX analysis of the OC, TC, and OTC fillers.

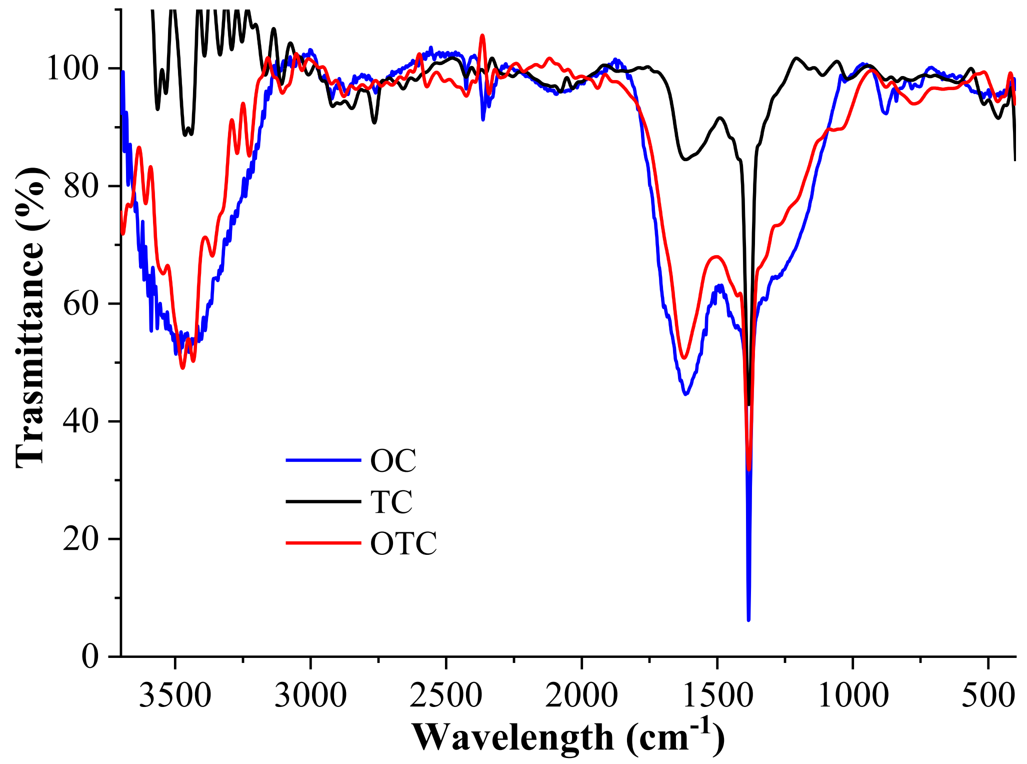

Figure S5. FTIR spectra of the OC, TC, and OTC fillers.

(Above 3000 cm^{-1}, the spectra for TC and OTC are particularly noisy)

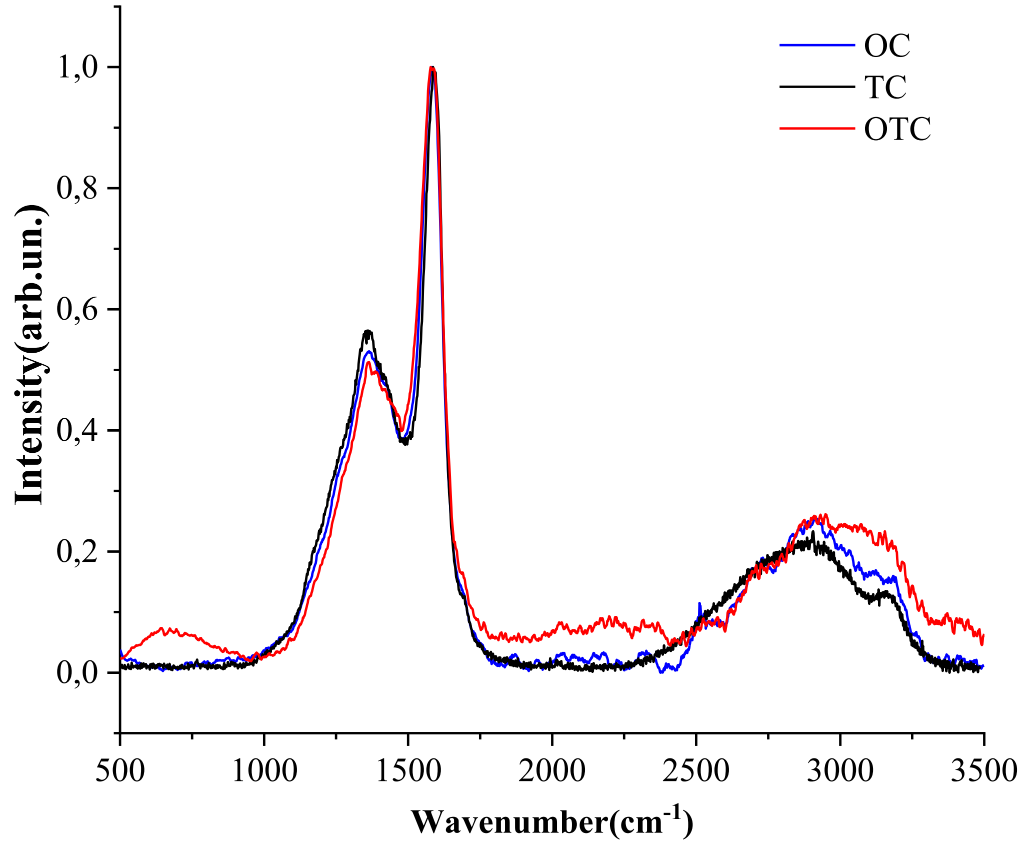

Figure S6. Raman spectra of the OC, TC, and OTC fillers.

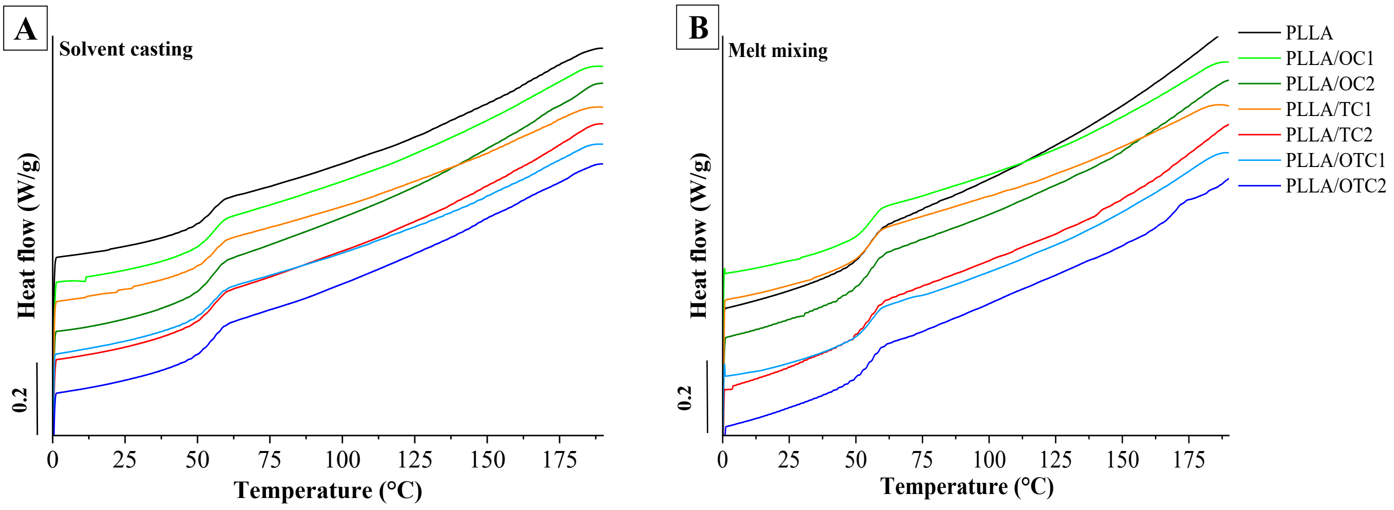

Figure S7. Cooling runs of PLLA samples obtained by **(A)** solvent casting and **(B)** melt mixing.
